# Traditional Chinese Medication Qiliqiangxin attenuates cardiac remodeling after acute myocardial infarction in mice

**DOI:** 10.1038/srep08374

**Published:** 2015-02-11

**Authors:** Lichan Tao, Sutong Shen, Siyi Fu, Hongyi Fang, Xiuzhi Wang, Saumya Das, Joost P. G. Sluijter, Anthony Rosenzweig, Yonglan Zhou, Xiangqing Kong, Junjie Xiao, Xinli Li

**Affiliations:** 1 Department of Cardiology, The First Affiliated Hospital of Nanjing Medical University, Nanjing 210029, China; 2 Regeneration and Ageing Lab and Experimental Center of Life Sciences, School of Life Science, Shanghai University, Shanghai 200444, China; 3 Cardiovascular Division of the Beth Israel Deaconess Medical Center and Harvard Medical School, Boston, MA 02215, USA; 4 Laboratory of Experimental Cardiology, University Medical Centre Utrecht, Utrecht 3508, The Netherlands

## Abstract

In a multicenter randomized double-blind study we demonstrated that Qiliqiangxin (QLQX), a traditional Chinese medicine, had a protective effect in heart failure patients. However, whether and via which mechanism QLQX attenuates cardiac remodeling after acute myocardial infarction (AMI) is still unclear. AMI was created by ligating the left anterior descending coronary artery in mice. Treating the mice in the initial 3 days after AMI with QLQX did not change infarct size. However, QLQX treatment ameliorated adverse cardiac remodeling 3 weeks after AMI including better preservation of cardiac function, decreased apoptosis and reduced fibrosis. Peroxisome proliferator-activated receptor-γ (PPARγ) was down-regulated in control animals after AMI and up-regulated by QLQX administration. Interestingly, expression of AKT, SAPK/JNK, and ERK was not altered by QLQX treatment. Inhibition of PPARγ reduced the beneficial effects of QLQX in AMI remodeling, whereas activation of PPARγ failed to provide additional improvement in the presence of QLQX, suggesting a key role for PPARγ in the effects of QLQX during cardiac remodeling after AMI. This study indicates that QLQX attenuates cardiac remodeling after AMI by increasing PPARγ levels. Taken together, QLQX warrants further investigation as as a therapeutic intervention to mitigate remodeling and heart failure after AMI.

Acute myocardial infarction (AMI) remains a leading cause of morbidity and mortality in most industrialized countries worldwide^1^. Advances in the treatment of AMI, such as early reperfusion therapy, have markedly reduced the mortality in patients with AMI^2^,^3^. However, the parallel increase of the prevalence and mortality from post-MI left ventricular (LV) remodeling and heart failure has emerged as a growing challenging health problem of concern^1^,^4^,^5^. Current therapeutic strategies to prevent LV remodeling post-MI are still limited^6^,^7^,^8^. Therefore, identification of additional therapeutic targets and treatment options to prevent adverse LV remodeling after AMI is highly needed^1^,^8^,^9^.

Qiliqiangxin (QLQX) is a specific traditional Chinese medicine extracted from 11 distinct herbs, including ginseng radix et rhizoma, astragali radix, aconiti lateralis radix preparata, semen descurainiae lepidii, salvia miltiorrhiza radix et rhizoma, alismatis rhizoma, cinnamomi ramulus, polygonati odorati rhizoma, periploca cortex, carthami flos, and citri reticulatae pericarpium^10^,^11^,^12^. QLQX was approved by the State Food and Drug Administration of China for the treatment of heart failure in 2004 and was included in the Chinese guidelines for management of heart failure in 2014^10^,^13^. QLQX has been reported to inhibit the development of cardiac hypertrophy, remodeling and dysfunction after transverse aorta constriction in mice, and to improve cardiac function in spontaneously hypertensive rats and after AMI in rats as well^14^,^15^,^16^,^17^,^18^. Recently, a multicenter randomized double-blind parallel-group placebo-controlled study from our group demonstrated that 12 weeks of treatment with QLQX reduced the levels of N-terminal pro-B-type natriuretic peptide (NT-proBNP) in 512 chronic heart failure patients^10^. In addition, New York Heart Association functional classification, left ventricular ejection fraction, 6-min walking distance, and quality of life also all improved with QLQX treatment^10^. All these observations suggest that QLQX has protective effects in heart disease^19^. Although the previous study in rat has provided some evidence for the beneficial effects of QLQX in cardiac remodeling after AMI^16^, the underlying mechanisms of any benefits in this setting, remains unclear.

Cardiac remodeling after AMI is a complex process with lots of continuous and overlapping events^9^. In an early phase, cardiac remodeling is a consequence of fibrotic repair of the necrotic area with scar formation^20^,^21^. After that, the remodeling process is driven by architectural rearrangements of the surviving myocardium including myocyte hypertrophy, myocardial fibrosis and progressive cardiac dysfunction^9^,^20^,^21^. The present therapeutic strategies include angiotensin-converting enzyme (ACE) inhibition, angiotensin type I receptor blocker therapy and beta-adrenergic blockade^9^,^20^,^21^. Besides that, cardiac resynchronization therapy (CRT) is also a therapeutic option^9^. However, despite these treatments, a substantial proportion of patients obatin limited benefits and have adverse outcomes^9^,^20^,^21^. Recent studies have provided some insights into molecular mechanisms responsible for cardiac remodeling such as activation of oxidant stress pathways, inflammatory pathways and matrix-metalloproteinase^9^,^20^,^21^. The peroxisome proliferation-activated receptors (PPARs) belong to the nuclear receptor superfamily of ligand-inducible transcription factors and have distinct functional domains including a C-terminal ligand-binding domain for ligand-dependent transactivation and an N-terminal transactivation domain for DNA binding^22^,^23^. The PPAR family is composed of three members: PPARα, PPARβ, and PPARγ^22^. Several lines of evidence suggest that activation of PPARγ protect cardiac remodeling after ischemia injury though their unique benefits are shadowed by the risk for fluide retention, weight gain, bone loss and congestive heart failure^22^,^23^,^24^,^25^,^26^. Despite controversial effects on the heart, PPARγ still receive most attention regarding its pronounced insulin sensitizing abilities and beneficial effects. Therefore, designing or identifying selective PPARγ modulators to retain the therapeutic effects without the unwanted adverse effects are highly desirable.

Here, we studied the early and late use of QLQX after AMI in mice and demonstrated that QLQX primarily attenuated cardiac remodeling after AMI by inducing PPARγ, a key regulator of cardiac energy metabolism after myocardial injury^24^,^25^,^26^, thereby preserving cardiac function, decreasing apoptosis and reducing cardiac fibrosis.

## Methods

This study was approved by the ethical committees of the Nanjing Medical University and all animal experiments were conducted under the guidelines on humane use and care of laboratory animals for biomedical research published by National Institutes of Health (No. 85-23, revised 1996).

### Establishment of AMI model

AMI was created by ligation the left anterior descending coronary artery (LAD) in mice as previous described^27^. Briefly, mice were fully anesthetized with 3% chloral hydrate while being mechanically ventilated with a rodent respirator. After the thoracic cavity was opened, the left coronary artery was ligated about 3–4 mm from the tip of the left auricle using 7–0 silk suture in AMI group while in the sham operated group, the needle was passed around the artery without ligation. Complete occlusion of the vessel was confirmed by the presence of myocardial blanching in the perfusion bed. After ligation, the chests of the mice were closed with a continuous 6–0 prolene suture, followed by a 4–0 polyester suture to close the skin and the mice were allowed to recover. In a subset of animals sacrificed at 3 days, before sacifice, 1 ml Evans blue (0.1 g/ml; BioSharp, Hefei, Anhui, China) was slowly injected into the abdominal aorta and the heart was immediately removed and infarct size was determined with triphenyltetrazolium chloride (TTC) staining (1%, Amresco, Radnor, Pennsylvania, USA). The ratio of infarct area to “area-at-risk” was calculated using Image J (NIH) from 6 sections throughout the heart.

### Animal groups

C57/BL6 male mice purchased from Nanjing University were used in this study at 10–12 weeks old. QLQX was provided by Shijiazhuang Yiling Pharmaceutical Co., Ltd. (Shijiazhuang, Hebei, China). As shown in our previous report^10^, to guarantee the quality and consistency of QLQX, the raw medicinal materials were of a certain variety, and their areas of origin, medicinal parts and processing methods were kept consistent. The stability of the product was verified by analyzing 10 batches of QLQX and fingerprints.

To determine the effects of QLQX on cardiac remodeling after AMI, mice were randomly divided into eight groups and were treated by intragastric administration as follows for 21 days: (i) sham operation + vehicle; (ii) sham operation + QLQX (0.25 g/kg/d); (iii) AMI + QLQX (0.25 g/kg/d); (iv) AMI + vehicle; (v) sham operation + QLQX (0.5 g/kg/d); (vi) AMI + QLQX (0.5 g/kg/d); (vii) sham operation + QLQX (0.75 g/kg/d); and (viii) AMI + QLQX (0.75 g/kg/d). To determine if QLQX provide beneficial effects through PPARγ, mice were randomly divided into eight groups and were treated as follows for 21 days: (i) sham operation + vehicle; (ii) sham operation + QLQX (0.5 g/kg/d); (iii) AMI + vehicle; (iv) AMI + QLQX (0.5 g/kg/d); (v) sham operation + Rosiglitazone (PPARγ activator at 1 mg/kg/d); (vi) AMI + Rosiglitazone (PPARγ activator 1 mg/kg/d); (vii) sham operation + T0070907 (PPARγ inhibitor 1 mg/kg/d); and (viii) AMI + T0070907 (PPARγ inhibitor 1 mg/kg/d). PPARγ activator and inhibitor were by intraperitoneal injection just after AMI.

To clarify whether QLQX affects the acute injury or post-MI remodeling, mice were either treated with QLQX or vehicle for the first 3 days after infarction or from day 3 to day 21 at a dose of 0.5 g/kg/d.

### Echocardiography

Echocardiography was performed in mice anesthetized with 1.5–2% isoflurane using a a Vevo 2100 (VisualSonics Inc, Toronto, Ontario, Canada) with a 30 MHz central frequency scan head. The following parameters were measured from M-mode images taken from the parasternal short-axis view at papillary muscle level: left ventricular internal dimension-diastole (LVIDd), left ventricular internal dimension-systole (LVIDs), left ventricular fractional shortening (FS) and left ventricular ejection fraction (EF).

### Histological examination

To visualize cardiomyocyte architecture, hematoxylin-eosin staining was performed. To assess the degree of fibrosis, sections were stained with Masson-Trichrome and scanned with computer-assisted video densitometry; images from at least 20 fields were analyzed for each heart, as previously described^28^. The fibrotic fraction was obtained by calculating the ratio of blue (fibrotic) to total myocardial area using Image J (NIH).

### Terminal deoxynucleotidyl transferase dUTP nick end labeling (TUNEL) staining

To detect apoptosis, TUNEL assays were performed using In Situ Cell Death Detection Kit according to the manufacturer's instructions (Roche, Mann-heim, German). Cell nuclei were counterstained with DAPI and the number of TUNEL-positive nuclei was counted. Ten fields (400 × magnification) from the AMI + vehicle and AMI + QLQX groups were used from each cryosection, using an average of three slides per heart.

### Western blotting

Cardiac tissues were lysed using RIPA buffer (Beyotime Institute of Biotechnology) containing a protease inhibitor cocktail (Sigma, St.louis, MO, USA). Equal amounts of protein were subjected to SDS-PAGE and transferred onto PVDF membranes. Standard western blot analysis was conducted using Transforming growth factor beta (TGF-β, 1:1000 dilution; Cell Signaling Technology, Boston, Massachusetts, USA), Mothers against decapentaplegic homolog 7 (SMAD7, 1:500 dilution; Santa cruz, San diego, California, USA), Matrix metalloproteinase-2 (MMP-2, 1:1000 dilution; Abcam,Cambrige,UK ),Matrix metallopeptidase 9 (MMP-9,1:1000 dilution; Abcam,Cambrige,UK), B-cell lymphoma 2 (Bcl-2, 1:1000 dilution; Cell Signaling Technology, Boston, Massachusetts, USA), Bcl-2-associated X protein (Bax, 1:1000 dilution; Cell Signaling Technology, Boston, Massachusetts, USA), Poly (ADP-ribose) polymerase (PARP, 1:1000 dilution; Cell Signaling Technology, Boston, Massachusetts, USA), Cleaved-PARP (1:1000 dilution; Cell Signaling Technology, Boston, Massachusetts, USA), Cleaved caspase 3 (1:1000 dilution; Cell Signaling Technology, Boston, Massachusetts, USA), Peroxisome proliferator-activated receptor alpha (PPARα, 1:1000 dilution; Abcam, Cambrige, UK), PPAR beta (PPARβ, 1:1000 dilution; Abcam, Cambrige, UK), PPAR gamma (PPARγ, 1:500 dilution; Abcam, Cambrige, UK), Peroxisome proliferator-activated receptor gamma coactivator 1-alpha (PGC-1α, 1:1000 dilution; NOVUS, Littleton, COLO, USA), Protein kinase B (Akt, 1:1000 dilution; Cell Signaling Technology), p-Akt (Ser473, 1:1000 dilution; Cell Signaling Technology, Boston, Massachusetts, USA), C-Jun N-terminal kinase (SAPK/JNK, 1:1000 dilution; Cell Signaling Technology, Boston, Massachusetts, USA), Phospho-SAPK/JNK (Thr183/Tyr185, 1:1000 dilution; Cell Signaling Technology, Boston, Massachusetts, USA), Extracellular signal regulated kinases (ERK, 1:1000 dilution; Cell Signaling Technology, Boston, Massachusetts, USA) andp-ERK (Thr202/Tyr204, 1:1000 dilution; Cell Signaling Technology, Boston, Massachusetts, USA). Glyceraldehyde 3-phosphate dehydrogenase antibody (GAPDH, 1:1000 dilution; Kangchen, Shanghai, China) was used as a loading control. After incubation with the appropriate secondary antibodies, signals were visualized using the ECL Plus Western blotting detection reagents (Bio-Rad) and the ChemiDoc XRS Plus luminescent image analyzer (Bio-Rad, Hercules, CA, USA). Densitometric analysis of band intensity was performed using Imagelab software (Bio-Rad, Hercules, CA, USA).

### Quantitative reverse transcription polymerase chain reactions (QRT-PCRs)

Total DNA-free RNA was extracted using miRNeasy Mini Kit (Qiagen, Hilden, Germany). cDNA synthesis was performed with Bio-Rad iScripTM cDNA Synthesis Kit (Bio-Rad, Hercules, CA, USA) according to the manufacturer's instructions in a reaction volume of 20 μl. For quantitative mRNA analysis, a template equivalent to 20 ng of total RNA was subjected to 40 cycles of quantitative PCR using the Takara SYBR Premix Ex TaqTM (TliRNaseH Plus, Takara,Tokyo, Japan) in the 7900HT Fast Real-Time PCR System. GAPDH was used for normalization. Relative mRNA expression was presented using the 2^−ΔΔCt^ method. Primer sequences (forward and reverse) used in the present study are listed as Supplemental Table 1.

### Statistical analysis

Data are expressed as mean ± SE. An independent-samples t-test, Chi-squared test or one-way ANOVA was conducted to evaluate the one-way layout data. If a significant difference was observed, Bonferroni's post-hoc test was conducted to identify groups with significant differences. P-values less than 0.05 were considered to be statistically significant. The overall survival rate was determined using Kaplan-Meier survival analysis and compared by log-rank test. All analyses were perfomed using SPSS 13.0 or GraphPad Prism 5.

## Results

### QLQX attenuates cardiac remodeling after AMI in mice

**Figure 1:**
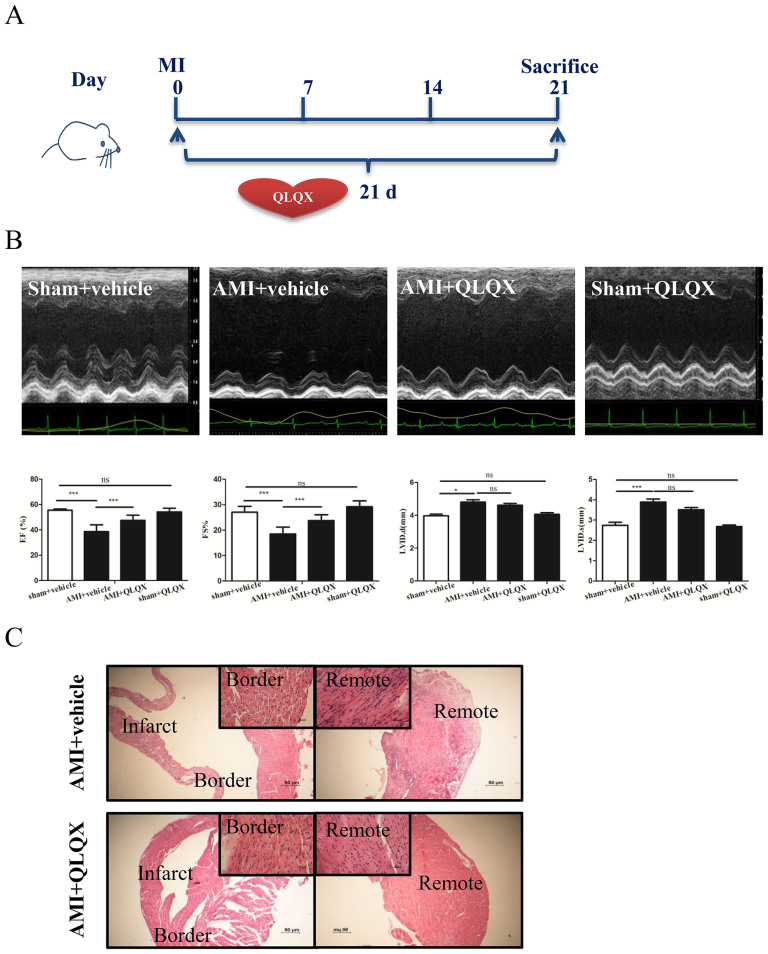
Qiliqiangxin (QLQX) improves cardiac function and preserves cardiomyocyte architecture. (A), A protocol schema for investigating the effects of QLQX in cardiac remodeling after acute myocardial infarction (AMI). Lichan Tao drew the mouse in figure 1A. (B), QLQX improves cardiac function including preserving left ventricular fractional shortening (FS) and left ventricular ejection fraction (EF). ns, not significant; *, P < 0.05; ***, P < 0.001; n = 10 per group. (C), QLQX preserves cardiomyocyte architecture.

We previously found that QLQX had a beneficial effects in heart failure patients in a multicenter randomized double-blind placebo-controlled study^10^. Here we sought to understand, whether QLQX improves cardiac remodeling after ischemic injury and the underlying mechanisms^10^,^19^. Mice were subjected to coronary artery ligation and subsequently treated with vehicle or QLQX for 21 days at one of three doses (0.25 g/kg/d, 0.5 g/kg/d, or 0.75 g/kg/d) (Figure 1A and Supplemental Figure 1). Cardiac function was examined by echocardiography (Fgure 1B and Supplemental Figure 1). Although QLQX had no effect on cardiac structure of function in sham operated animals, when delivered at 0.5 g/kg/d or 0.75 g/kg/d, it consistently improved cardiac function including improvement in EF and FS (Figure 1B and Supplemental Figure 1A). The lowest dosage of QLQX (0.25 g/kg/d) did not significantly affect cardiac function (Supplemental Figure 1B). Based on these findings, QLQX was used at a dosage of 0.5 g/kg/d, which is comparable to the dosage of QLQX applied to human in clinic, in the subsequent studies described below.

**Figure 2:**
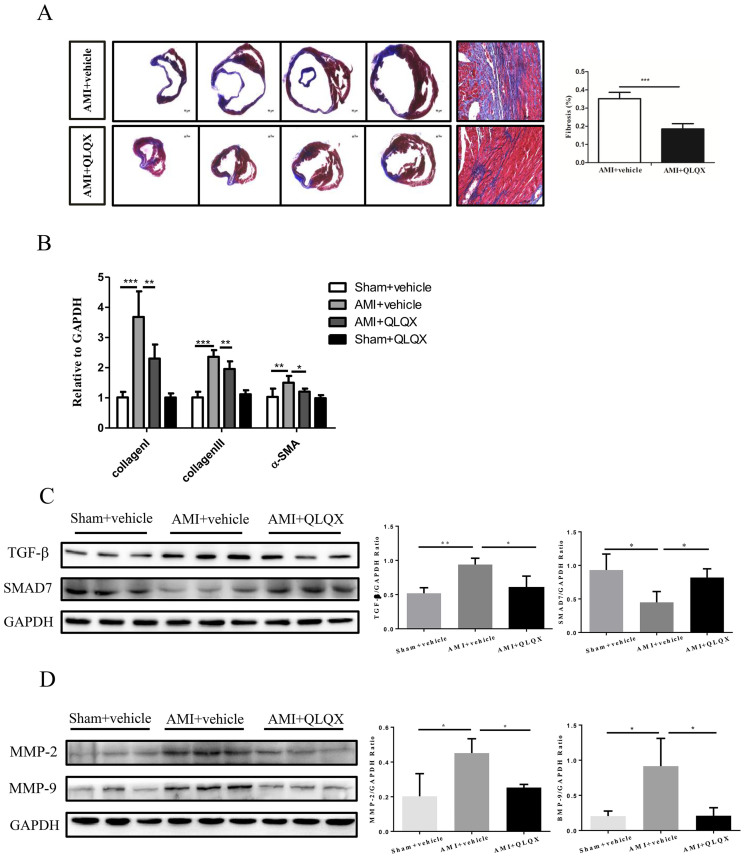
Qiliqiangxin (QLQX) decreases cardiac fibrosis. (A), QLQX attenuates cardiac fibrosis. ***, P < 0.001. n = 6 per group. (B), QLQX decreases mRNA levels of collagen I, collagen III and α-SMA. *, P < 0.05; **, P < 0.01; ***, P < 0.001. n = 6 per group. (C), QLQX decreases the expression level of TGF-β1 and increases that of Smad7. *, P < 0.05; **, P < 0.01. n = 3 per group. (D), QLQX decreases the expression levels of MMP-2 and MMP-9. *, P < 0.05. n = 3 per group. Cropped blots were used here and the full-length gels were included in the supplementary information.

**Figure 3:**
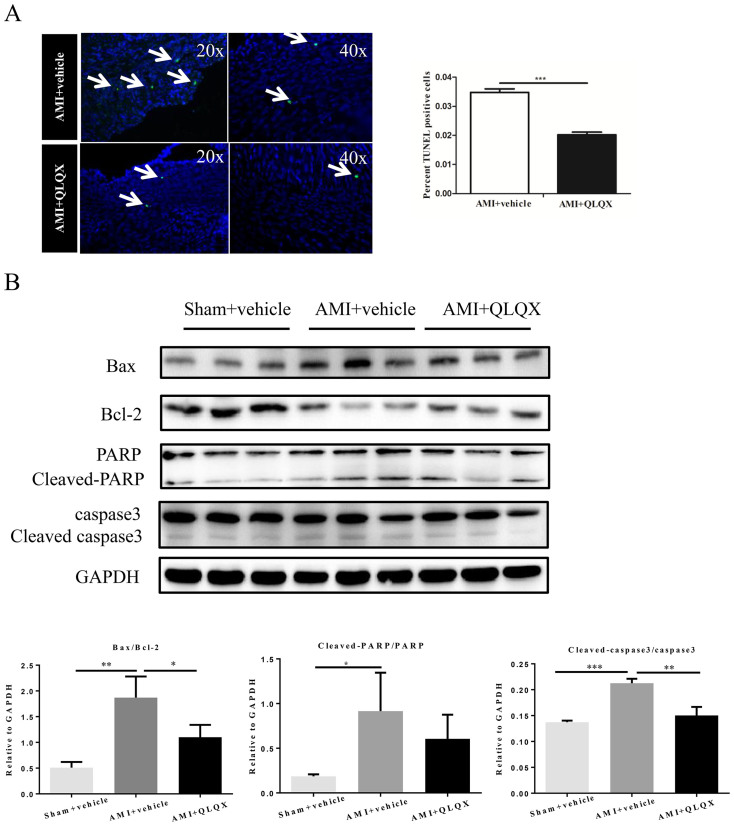
Qiliqiangxin (QLQX) attenuates cardiac apoptosis. (A), QLQX decreases cardiac apoptosis. ***, P < 0.001. n = 6 per group. (B), QLQX decreases the ratio of Bax/Bcl-2, cleaved PARP/PARP and cleaved caspase-3/ caspase-3. *, P < 0.05; **, P < 0.01; ***, P < 0.001. n = 3 per group. Cropped blots were used here and the full-length gels were included in the supplementary information.

As shown in Figure 1C, the surviving myocardial cells in the infarct border zones of vehicle-treated animals exhibited greater irregularity and disarray in comparison to QLQX-treated animals in which myocardial cells manifested a more organized alignment. These data suggest that cardiomyocyte architecture in AMI was better preserved in QLQX-treated animals. Cardiac fibrosis is a well-known feature of cardiac remodeling after AMI^29^,^30^. Masson-Trichrome staining revealed that cardiac fibrosis was significantly attenuated in mice receiving QLQX as compared to vehicle after AMI (Figure 2A), which was consistent with the decreased levels of collagen I, collagen III and α-SMA (Figure 2B). As TGF-β1/Smad7 signaling and MMP-2/9 are well recognized as the contributors for cardiac remodeling after AMI^9^,^20^,^21^, we also checked their expression levels after QLQX treatment. As shown in Figure 2C and 2D, TGF-β1, MMP-2 and MMP-9 were downregulated and Smad7 was upregulated in QLQX-treated mice after AMI, which might be partly responsible for the effects of QLQX treatment. Cardiac apoptosis, another feature of ventricular remodeling after AMI^6^, was also significantly decreased in the QLQX treated animals as assessed by TUNEL staining (Figure 3A). Immunoblotting revealed that the ratio of pro-apoptotic molecule, Bax, to anti-apoptotic molecule, Bcl-2, was decreased in QLQX-treated mice after AMI (Figure 3B). Similarly, the ratios of cleaved PARP to PARP and cleaved caspase-3 to caspase-3 after AMI were also decreased in the QLQX treatment group (Figure 3B) compared to vehicle treated animals. Collectively, these data confirm that QLQX attenuates cardiac remodeling after AMI in mice by decreasing fibrosis and apoptosis.

### PPARα and PPARγ are increased with QLQX treatment post-MI

**Figure 4:**
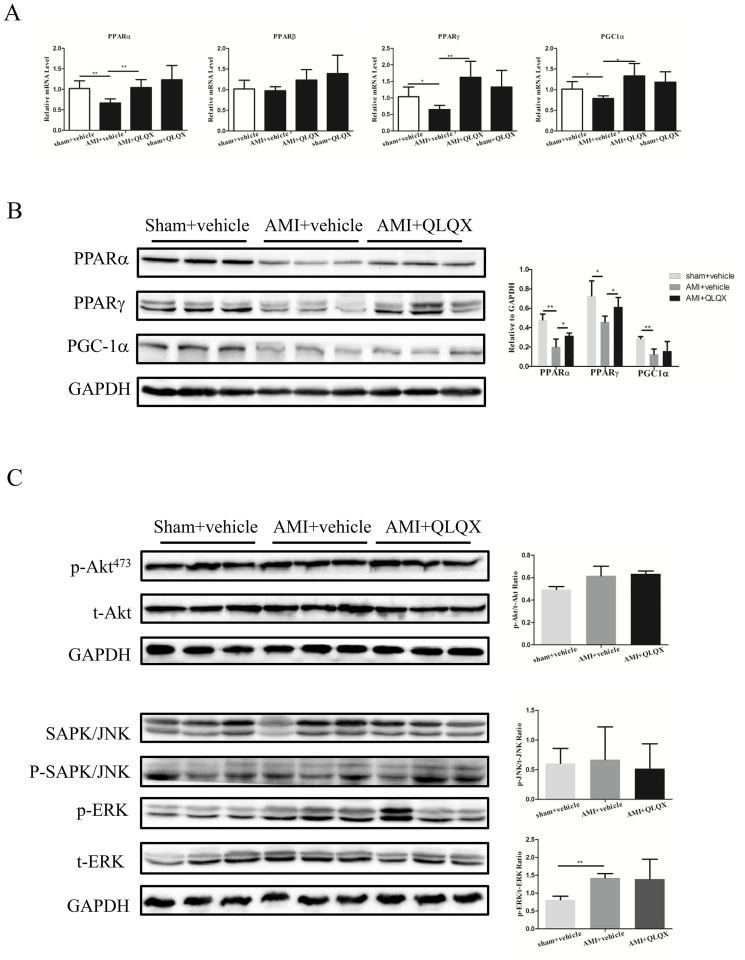
Qiliqiangxin (QLQX) increases PPARα and PPARγ. (A), QLQX increases PPARα, PPARγ, and PGC1α at the mRNA level. *, P < 0.05; **, P < 0.01. n = 6 per group. (B), QLQX increases PPARα and PPARγ at the protein level. *, P < 0.05; **, P < 0.01. n = 3 per group. (C), QLQX does not regulate AKT, SAPK/JNK, and ERK pathways. ns, not significant; **, P < 0.01. n = 3 per group. Cropped blots were used here and the full-length gels were included in the supplementary information.

Derangement in myocardial fuel utilization is another hallmark of cardiac remodeling after AMI^1^,^26^,^31^. PPAR nuclear receptor transcription factors are key regulators of cellular metabolism in the heart^26^,^31^,^32^. Interestingly, mRNA levels of PPARα, PPARγ, and PGC-1α were all downregulated in vehicle-treated animals after AMI and these decreases were prevented by QLQX treatment after AMI (Figure 4A), while PPARβ was not altered. Immunoblotting showed that the levels of PPARα and PPARγ protein were also increased in QLQX-treated mice after AMI, while PGC-1α protein levels were not affected by QLQX (Figure 4B). AKT, SAPK/JNK, and ERK were not different between vehicle and QLQX treatment in the AMI model (Figure 4C), suggesting that these signaling pathways were not involved in QLQX-mediated improvement in cardiac remodeling after AMI. These data suggest that induction of PPARα and PPARγ could contribute to the beneficial effects of QLQX in attenuating cardiac remodeling after AMI.

### QLQX attenuates cardiac remodeling after AMI via PPARγ

To further interrogate the contribution of PPARα and PPARγ to the beneficial effects of QLQX after AMI, we investigated the effects of activation of PPARα (WY-14643, 1 mg/kg/d) or PPARγ (Rosiglitazone, 1 mg/kg/d) on cardiac function 21 days after AMI. The effects of WY-14643 and Rosiglitazone were confirmed by examination of PPARα and PPARγ protein levels and their downstream target genes (Supplemental Figure 2A–D). Interestingly, the PPARγ activator mimicked the effects of QLQX, improving cardiac function (including restoring EF and FS), while the PPARα activator had no effect on cardiac function (Supplemental Figure 2E–G), indicating that PPARα is unlikely to mediate the effects of QLQX on cardiac remodeling after AMI.

**Figure 5:**
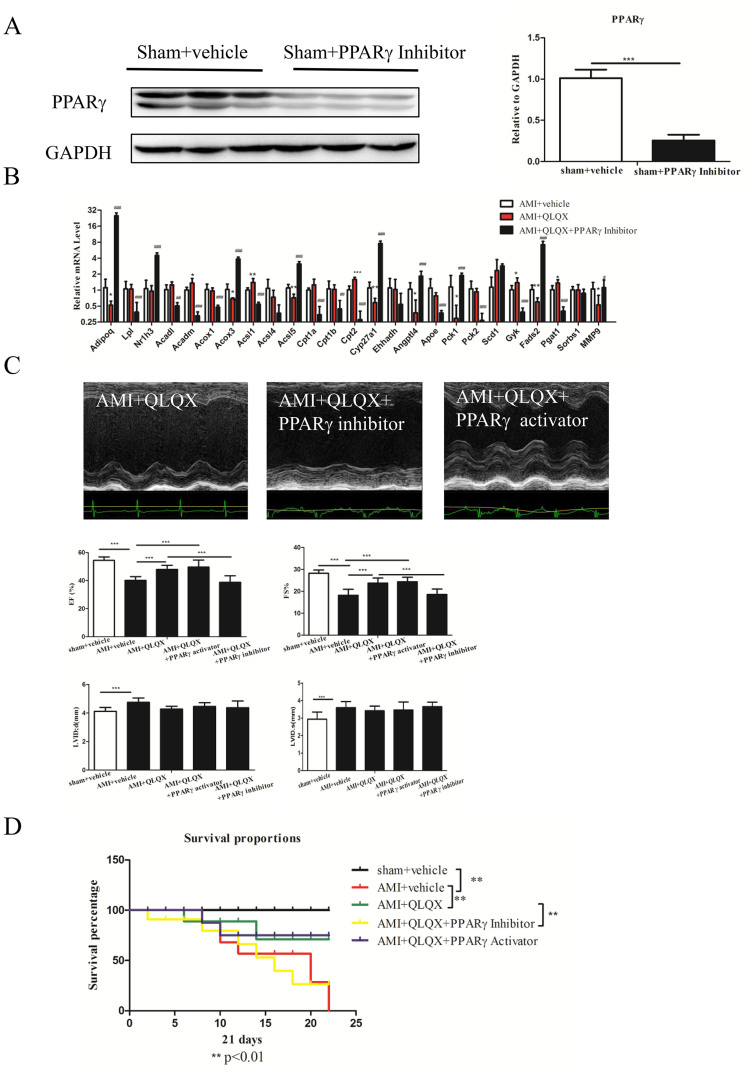
Qiliqiangxin (QLQX) improves cardiac function and survival rate after acute myocardial infarction (AMI) via increasing PPARγ. (A), PPARγ inhibition decreases the expression of PPARγ. ***, P < 0.001. n = 3 per group. Cropped blots were used here and the full-length gels were included in the supplementary information. (B), PPARγ inhibition regulates its downstream target genes. *, P < 0.05; **, P < 0.01; ***, P < 0.001. n = 6 per group. (C), PPARγ inhibitors abolish the beneficial effects of QLQX in improving cardiac function while PPARγ activators failed to provide any additional improvement in cardiac function in the presence of QLQX. ***, P < 0.001. n = 6 per group. (D), QLQX improves the survival rate after AMI and combining PPARγ inhibitors fails to provide additional beneficial effects. **, P < 0.01. n = 6 per group.

To determine if PPARγ is necessary for the benefits of QLQX after AMI, we treated animals with T0070907, an inhibitor of PPARγ (1 mg/kg/d), in combination with QLQX after AMI. The effects of PPARγ inhibitor were confirmed by the decreased PPARγ expression levels (Figure 5A). The PPARγ inhibitor did not affect baseline cardiac physiological parameters including EF, FS, LVIDd, and LVIDs (Supplemental Figure 3). However, T0070907 reversed the effects of QLQX treatment on many PPARγ target genes (Figure 5B). Treatment with the PPARγ inhibitor also completely abolished the beneficial effects of QLQX on cardiac function (assessed by EF and FS) after AMI, (Figure 5C), suggesting that PPARγ activation is necessary for QLQX's beneficial effects in attenuating cardiac remodeling after AMI. Moreover, PPARγ activators failed to provide any additional improvement in cardiac function in the presence of QLQX (Figure 5C). Finally, we found a survival benefit after MI for mice treated with QLQX, an effect that was abolished with the PPARγ inhibitors, but not further enhanced with the addition of PPARγ activators (Figure 5D). These experiments provide direct support for our hypothesis that PPARγ is necessary for QLQX-mediated improvement of cardiac function after AMI. In addition, the lack of additional benefit of PPARγ activators over QLQX in attenuating cardiac remodeling suggest a ‘plateau' effect for the benefits of QLQX via PPARγ and a relatively small contribution for PPARγ-independent pathways.

**Figure 6:**
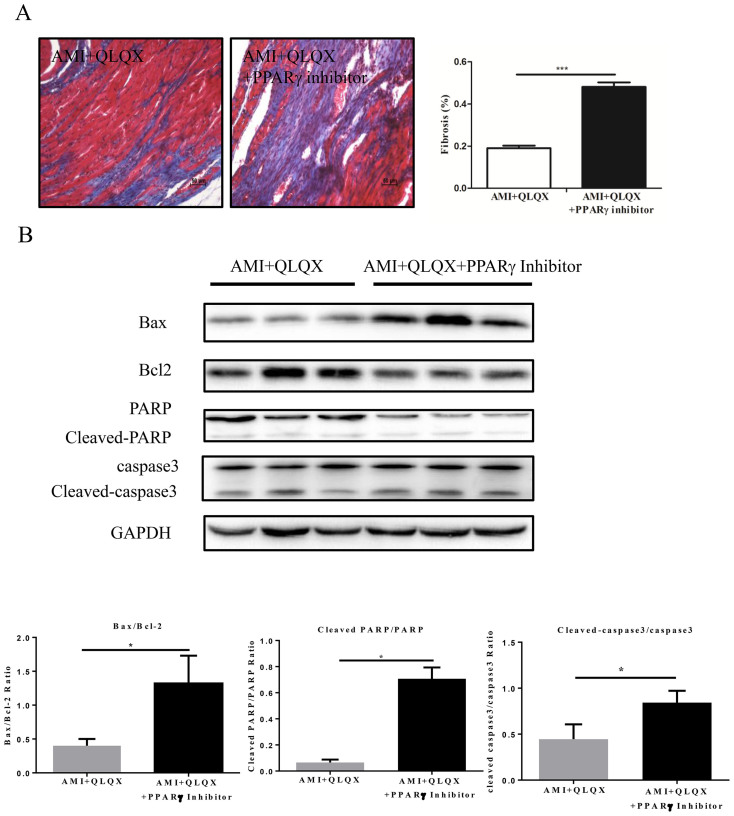
Qiliqiangxin (QLQX) attenuates cardiac fibrosis and apoptosis via PPARγ. (A), PPARγ inhibitors abolish the effects of QLQX in decreasing cardiac fibrosis. ***, P < 0.001. n = 6 per group. (B), PPARγ inhibitors abolish the effects of QLQX in decreasing apoptosis as indicated by the ratio of Bax/Bcl-2, cleaved PARP/PARP and cleaved caspase-3/ caspase-3. *, P < 0.05. n = 3 per group. Cropped blots were used here and the full-length gels were included in the supplementary information.

In addition to its contribution to improved cardiac function post-MI, PPARγ likely mediated the beneficial effects of QLQX on fibrosis post-MI, because the PPARγ inhibitor blocked the effects of QLQX on cardiac fibrosis as determined by Masson-Trichrome staining (Figure 6A). Finally, the anti-apoptotic effects of QLQX also appear mediated via PPARγ, since multiple apoptotic indices including the ratios of Bax/Bcl-2, cleaved PARP/PARP and cleaved caspase-3/caspase-3 were also shifted to a pro-apoptotic state when QLQX treatment was combined with the PPARγ inhibitor (Figure 6B).

### QLQX does not affect acute ischemic injury

**Figure 7:**
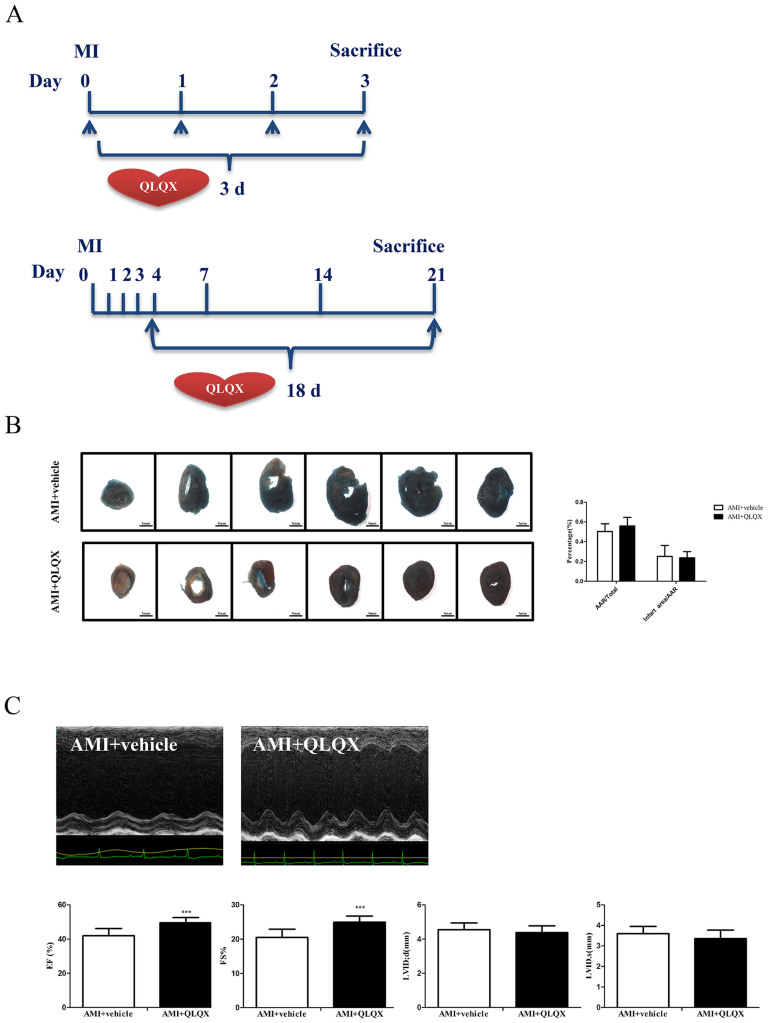
Qiliqiangxin (QLQX) provides beneficial effects after acute myocardial infarction (AMI) in remodeling phase but not in acute stage. (A), Protocol schemata for investigating the effects of QLQX in either acute or remodeling stage after AMI. (B), QLQX does not affect the infarction size in acute stage. n = 10 per group. (C), QLQX improves cardiac function when treating from chronic phase. ***, P < 0.001; n = 10 per group.

Since initial infarct size is a critical determinant of subsequent ventricular remodeling^33^, we sought to clarify the benefits of QLQX treatment were mediated by an initial cardioprotective reduction in infarct size or effects on subsequent ventricular remodeling. Mice were treated with QLQX or vehicle during the first three days after coronary ligation^34^ or from the third day on (Figure 7A). Importantly, QLQX had no effect on initial infarct size as determined by measuring the percentage of myocardial ischemic infarction size/area-at-risk (AAR) based on TTC and Evans Blue staining (Figure 7B). In contrast, treatment with QLQX beginning on the third day post-AMI, significantly improved cardiac function, as reflected by FS and EF (Figure 7C). Taken together, these experiments demonstrate that QLQX specifically mitigates adverse ventricular remodeling rather than affecting initial infarct size.

## Discussion

Adverse remodeling after AMI is a big challenge worldwide^2^,^9^. Although substantial progress has been achieved in lowering acute mortality rates after AMI, much of the ongoing morbidity and mortality relates to chronic adverse remodeling that occur after infarction^1^. Currently, the treatments available for this chronic phase are limited. Our previous randomized controlled trial has proved that QLQX provides benefits in patients with chronic heart failure^10^. To better understand these benefits and their mechanisms, we studied the effects of QLQX in a mouse model of AMI. Several observations were particularly noteworthy. First, we found that QLQX improved cardiac function including preserving EF and FS 21 days after AMI despite having no effect on initial infarct size. Second, apoptosis was decreased and cardiomyocyte architecture was better-preserved with QLQX treatment. Third, cardiac fibrosis was attenuated with QLQX treatment. Finally, the survival rate post-MI was improved by QLQX. Taken together, these data demonstrate that QLQX attenuates cardiac remodeling after AMI in mice, culminating in better preserved cardiac structure and function.

Cardiac remodeling is often associated with events that occur weeks and months after an AMI, and its consequences are invariably related to the initial size of the associated infarction^1^,^2^,^9^,^30^. The mice used in this study were treated with QLQX after coronary artery ligation, which mimics the situation encountered in the clinic. To determine whether QLQX affected initial infarct size, mice were treated with QLQX for 3 days immediately after coronary ligation and infarct size relative to the non-perfused (area-at-risk) was determined. We found no difference in initial infarct size in QLQX treated animals, suggesting its benefits occur predominantly during remodeling. To further confirm this hypothesis, mice were treated with QLQX for eighteen days beginning on the third day after coronary ligation. In this cohort, QLQX improved EF and FS comparably to mice treated for a full 21 days beginning immediately after coronary ligation. Taken together, these data provide strong support for the hypothesis that QLQX mitigates adverse remodeling without affecting the initial infarct size. This finding underscores the potential translational implications of these findings since drug delivery hours to days after infarction is far more feasible in the clinical setting. However, further investigation would be necessary to determine whether QLQX provides incremental benefits over the current standard of care for post-AMI patients.

To investigate the mechanisms underlying the beneficial effects of QLQX in AMI remodeling, several well-known signaling pathways including AKT, SAPK/JNK, and ERK were interrogated but not found to be altered with QLQX treatment^4^,^6^,^9^. Instead, we found that PPARγ is increased by QLQX treatment and appears to be responsible for the beneficial effects of QLQX in ventricular remodeling as supported by several lines of evidence. First, the expression of PPARγ is downregulated in AMI but expression is maintained after AMI by QLQX treatment at both the mRNA and protein levels. Secondly, T0070907, an inhibitor of PPARγ abolished the protective effects of QLQX on cardiac remodeling suggesting PPARγ is necessary for QLQX's benefits. PPARγ activation was also sufficient to improve cardiac function after AMI. Moreover, PPARγ activators failed to provide additional improvement in cardiac function in the presence of QLQX, suggesting the two work predominantly through the same pathway which is fully activated with either treatment alone. Accumulating evidence has suggested that PPARγ activators could protect the heart from adverse remodeling after ischemia injury^31^,^35^,^36^. However, PPARγ activators are currently contraindicated in heart failure due to an increased incidence of fluid retention and edema^27^,^28^. In addition, there are concerns that PPARγ activators paradoxically increased the incidence of AMI in diabetic patients^37^,^38^. We did not observe signs of edema with QLQX treatment (data not shown) in this study, but further investigation would be needed to determine if QLQX shares any of the adverse effects seen with PPARγ activators. It's also possible that QLQX might provide a new way to mediate the benefits of PPARγ activation without the adverse effects.

Several limitations of the present study should be highlighted. Firstly, as myocardial tissue contains several types of cells, it would be interesting to determine in which cell type the alteration of PPARγ occurs. Secondly, as QLQX contains 11 distinct active components, which compound(s) in QLQX activate PPARγ to protect cardiac remodeling warrants further investigation.

Taken together, the present study highlights the therapeutic effects of QLQX in AMI remodeling. Our pharmacological experiments suggest that QLQX attenuates cardiac remodeling after AMI by increasing PPARγ. Genetic approaches such as the generation of cardiomyocyte-specific PPARγ knockout mice will be of interest for future studies to fully reveal the role of PPARγ activation in the therapeutic effects of QLQX^39^. Although the exact active ingredients of QLQX responsible for the beneficial effects of QLQX need to be determined in the future^19^, our data present here combined with our previous clinical trial data suggest that QLQX warrants further investigation as a therapeutic intervention for a range of post-AMI and heart failure patients^10^.

## Supplementary Material

Supplementary Information Supplementary Material
