# Supplementary material for: Traditional Chinese Medication Qiliqiangxin attenuates cardiac remodeling after acute myocardial infarction in mice
Source: Sci Rep. 2015 Feb 11;5:8374. doi: 10.1038/srep08374 (PMC4648480; doi:10.1038/srep08374)

Traditional Chinese Medication Qiliqiangxin attenuates cardiac remodeling after acute myocardial infarction in mice

Lichan Tao, Sutong Shen, Siyi Fu, Hongyi Fang, Xiuzhi Wang, Saumya Das, Joost PG Sluijter, Anthony Rosenzweig, Yonglan Zhou, Xiangqing Kong, Junjie Xiao, Xinli Li

SUPPLEMENTAL MATERIAL

Supplemental Table 1 Primers used in this study

Gene	Species	Forward Primer	Reverse Primer
PPAR alpha	mouse	AGAGCCCCATCTGTCCTCTC	ACTGGTAGTCTGCAAAACCAAA
PPAR delta	mouse	TCCATCGTCAACAAAGACGGG	ACTTGGGCTCAATGATGTCAC
PPAR gamma	mouse	TCCTGTAAAAGCCCGGAGTAT	GCTCTGGTAGGGGCAGTGA
PGC-1alpha	mouse	TATGGAGTGACATAGAGTGTGCT	CCACTTCAATCCACCCAGAAAG
PGC-1beta	mouse	CTTGGCTGCGCTTACGAAGA	GAAAGCTCGTCCACGTCAGAC
Adipoq	mouse	ATGACGACTGCCATCCTAGAG	GCTCCCTAAAGAGCTGGGG
LPL	mouse	GGTTGCGCGTAGAGAGGATG	CTCACGCTCTGACATGCCTTC

Nr1h3	mouse	CTCAATGCCTGATGTTTCTCCT	TCCAACCCTATCCCTAAAGCAA
Acadl	mouse	TCTTTTCCTCGGAGCATGACA	GACCTCTCTACTCACTTCTCCAG
Acadm	mouse	AGGGTTTAGTTTTGAGTTGACGG	CCCCGCTTTTGTCATATTCCG
Acox1	mouse	TAACTTCCTCACTCGAAGCCA	AGTTCCATGACCCATCTCTGTC
Acox3	mouse	ACCGGAAGAAAAAGACAGTGC	GAGGCTCTTGCTCGGTAGG
Acsl1	mouse	TGCCAGAGCTGATTGACATTC	GGCATACCAGAAGGTGGTGAG
Acsl4	mouse	CTCACCATTATATTGCTGCCTGT	TCTCTTTGCCATAGCGTTTTTCT
Acsl5	mouse	TCCTGACGTTTGGAACGGC	CTCCCTCAATCCCCACAGAC
cpt1a	mouse	CTCCGCCTGAGCCATGAAG	CACCAGTGATGATGCCATTCT
cpt1b	mouse	GCACACCAGGCAGTAGCTTT	CAGGAGTTGATTCCAGACAGGTA
cpt2	mouse	CAGCACAGCATCGTACCCA	TCCCAATGCCGTTCTCAAAAT
cyp27a1	mouse	CCAGGCACAGGAGAGTACG	GGGCAAGTGCAGCACATAG
Ehhadh	mouse	ATGGCTGAGTATCTGAGGCTG	GGTCCAAACTAGCTTTCTGGAG

Fads2	mouse	AAGGGAGGTAACCAGGGAGAG	CCGCTGGGACCATTTGGTAA
Scd1	mouse	ACTGTGGAGACGTGTTCTGGA	ACGGGTGTCTGGTAGACCTC
Angptl4	mouse	CATCCTGGGACGAGATGAACT	TGACAAGCGTTACCACAGGC
Sorbs1	mouse	CCCCGTCTGAGGTAATAGTTGT	CATCGAGTGTCGGGAAGGTC
Apoa1	mouse	GGCACGTATGGCAGCAAGAT	CCAAGGAGGAGGATTCAAACTG
Apoc3	mouse	TACAGGGCTACATGGAACAAGC	CAGGGATCTGAAGTGATTGTCC
Apoe	mouse	CTGACAGGATGCCTAGCCG	CGCAGGTAATCCCAGAAGC
PECAM	mouse	ACGCTGGTGCTCTATGCAAG	TCAGTTGCTGCCCATTCATCA
GYK	mouse	TGAACCTGAGGATTTGTCAGC	CCATGTGGAGTAACGGATTTCG
Dgat1	mouse	TCCGTCCAGGGTGGTAGTG	TGAACAAAGAATCTTGCAGACGA
Pck2	mouse	ATGGCTGCTATGTACCTCCC	GCGCCACAAAGTCTCGAAC
MMP9	mouse	CTGGACAGCCAGACACTAAAG	CTCGCGGCAAGTCTTCAGAG
GAPDH	mouse	AGGTCGGTGTGAACGGATTTG	TGTAGACCATGTAGTTGAGGTCA

Supplemental Figure Legends

Supplemental Figure 1 Qiliqiangxin (QLQX) improves cardiac function at a dosage of 0.75g/kg/d while a lower dosage of QLQX (0.25g/kg/d) failed to exhibit the beneficial effect

ns, not significant; **, P<0.01; ***, P<0.001. n=10 per group.

Supplemental Figure 2 PPARγ activation but not PPARα improves cardiac function in AMI

A, The PPARα activator increases the expression of PPARα. **, P<0.01. n=3 per group. Cropped blots were used here and the full-length gels were included in the supplementary information. **B,** The PPARγ activator increases the expression of PPAR. ***, P<0.001. n=3 per group. Cropped blots were used here and the full-length gels were included in the supplementary information. **C,** PPARα activation regulates its downstream target genes. *, P<0.05; **, P<0.01; ***, P<0.001. n=6 per group. **D,** PPARγ activation regulates its downstream target genes. *, P<0.05; **, P<0.01; ***, P<0.001. n=6 per group. **E,** Representive echocardiographic images. **F,** PPARα activation does not affect cardiac function after AMI. n=6 per group. **G,** PPARγ activation improves cardiac function after AMI. ***, P<0.001. n=6 per group.

Supplemental Figure 3 PPARγ inhibitor does not affect the physiological parameters of cardiac function

A, Left ventricular ejection fraction (EF). **B，**left ventricular fractional shortening (FS). **C,** Left ventricular internal dimension-diastole (LVIDd). **D,** Left ventricular internal dimension-systole (LVIDs). n=6 per group.

Supplemental Figure 1

A

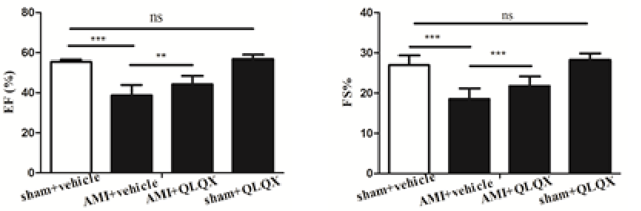

B

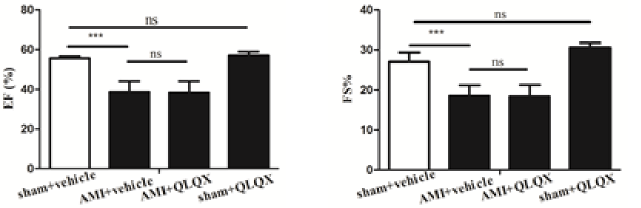

Supplemental Figure 2

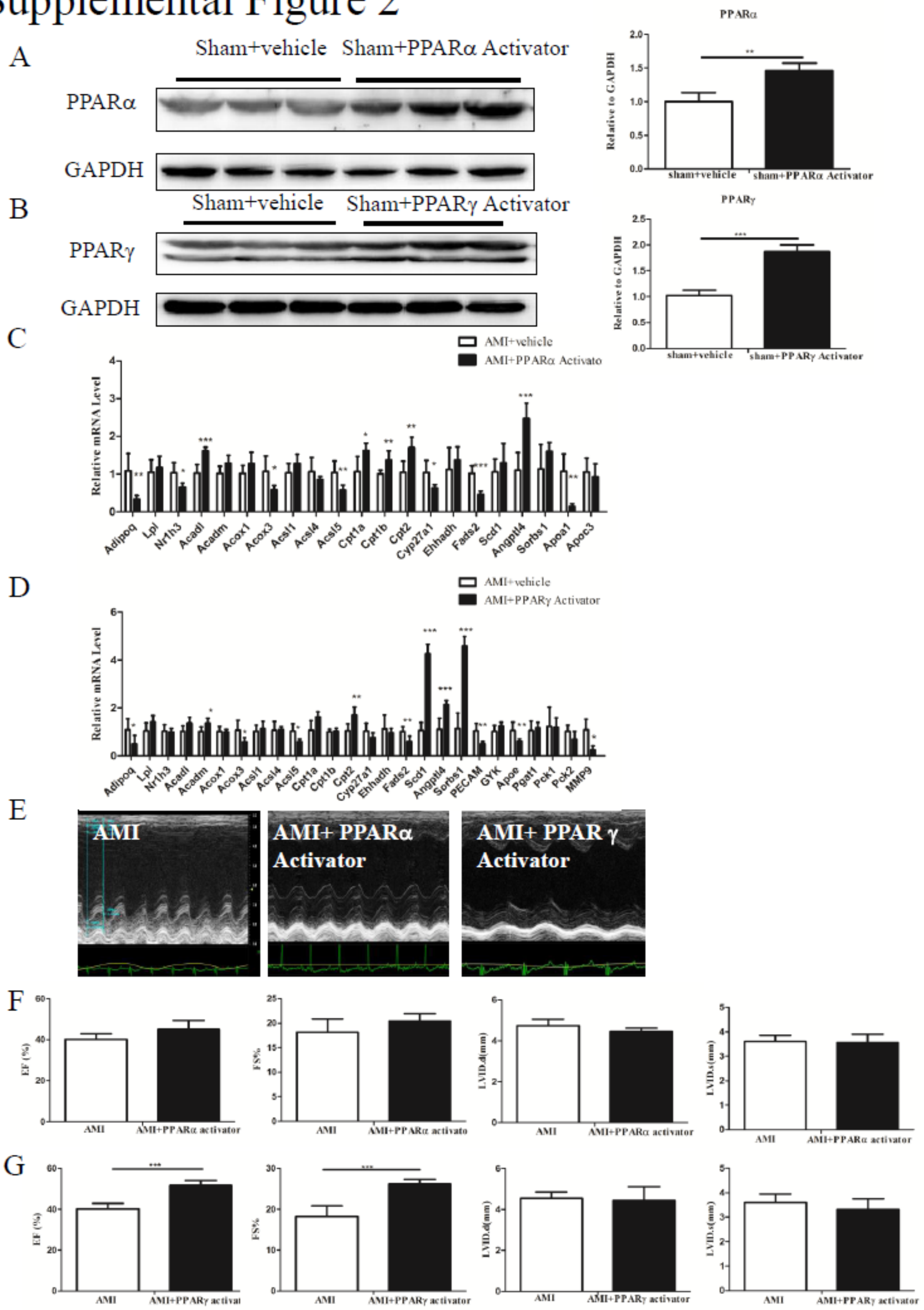

Supplemental Figure 3

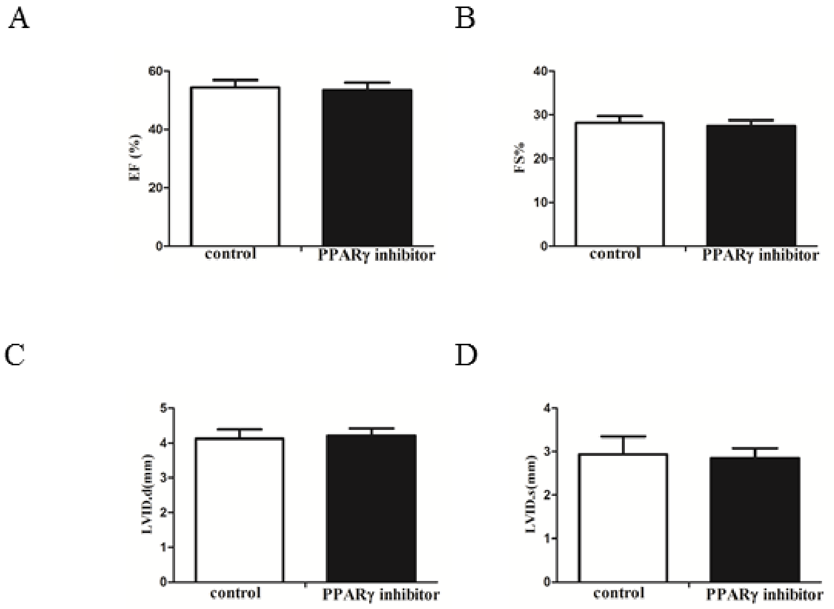

Full-length gels

Figure 2C

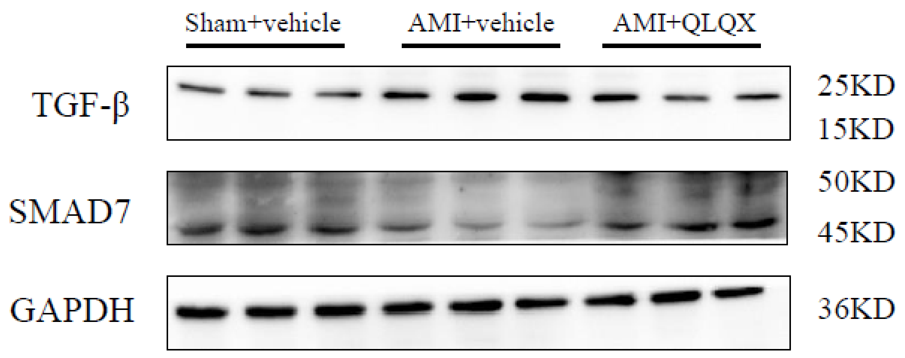

Figure 2D

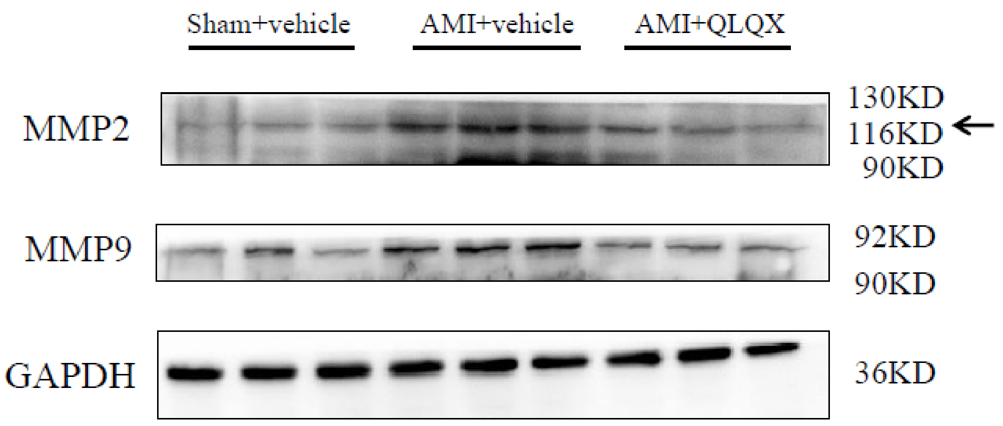

Figure 3B

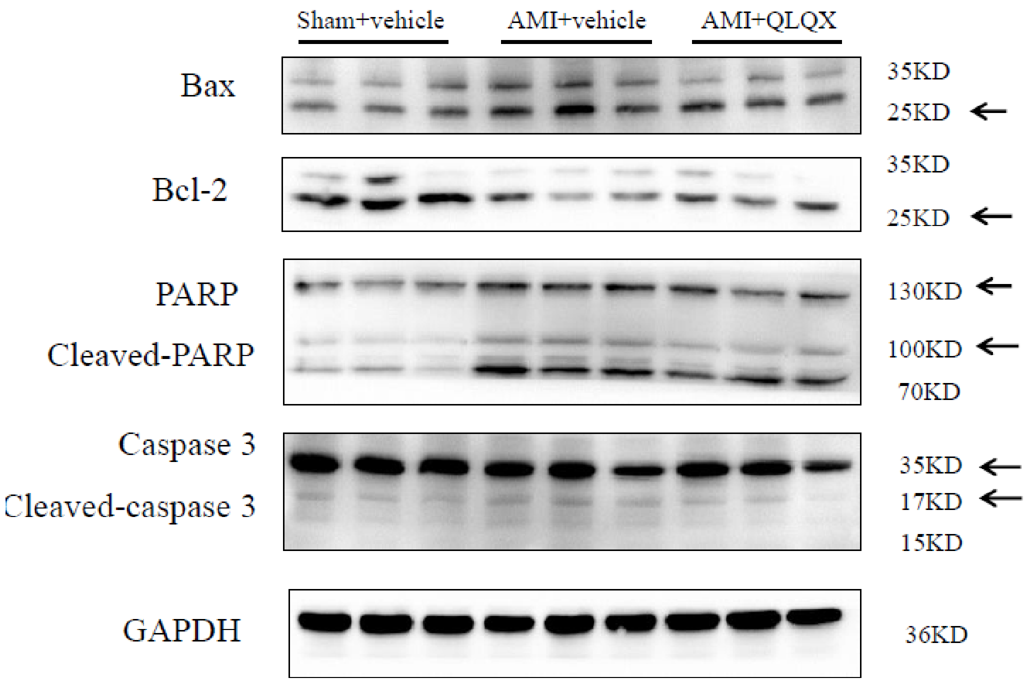

Figure 4 B

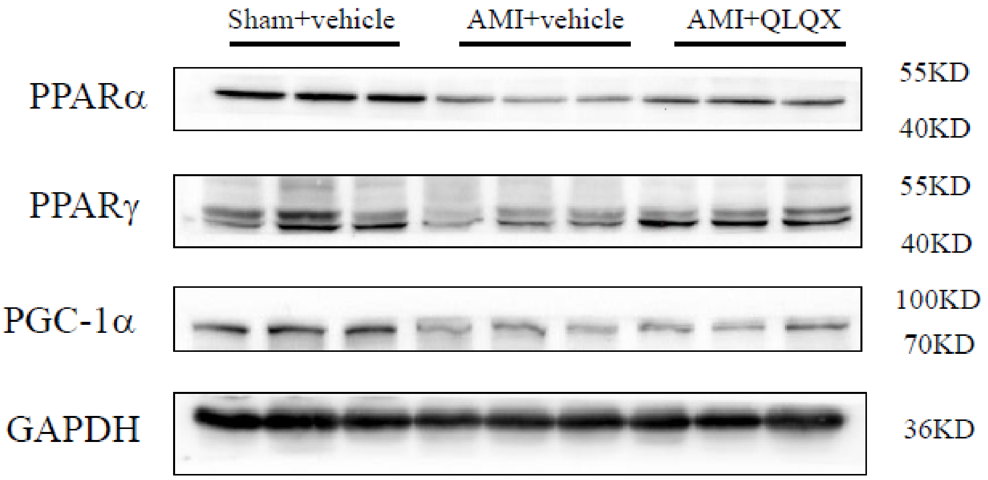

Figure 4 C

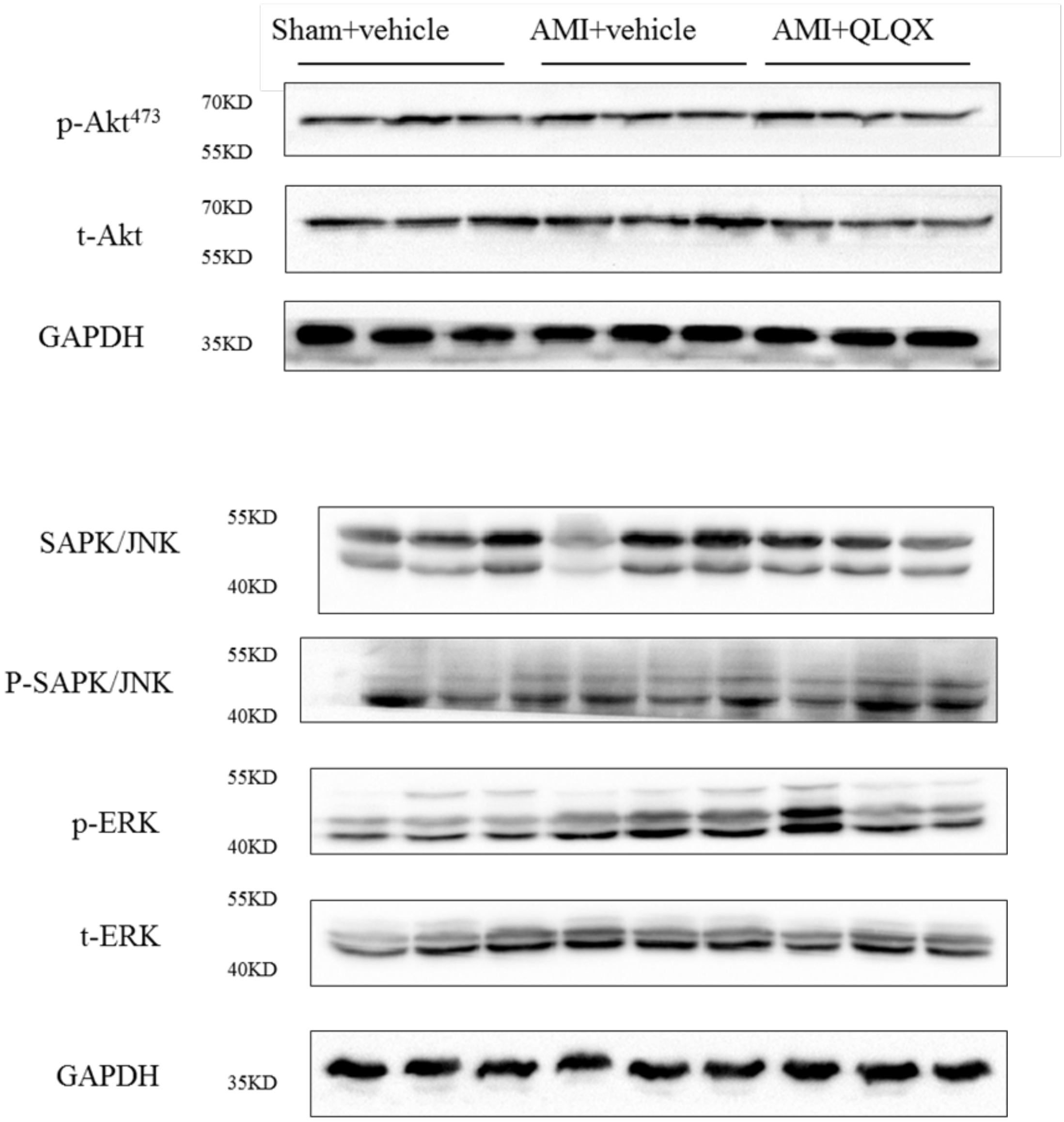
Sham+vehicle
AMI+vehicle
AMI+QLQX
p-Akt473
70KD
55KD
t-Akt
70KD
55KD
GAPDH
35KD
SAPK/JNK
55KD
40KD
P-SAPK/JNK
55KD
40KD
p-ERK
55KD
40KD
t-ERK
55KD
40KD
GAPDH
35KD

Figure 5A

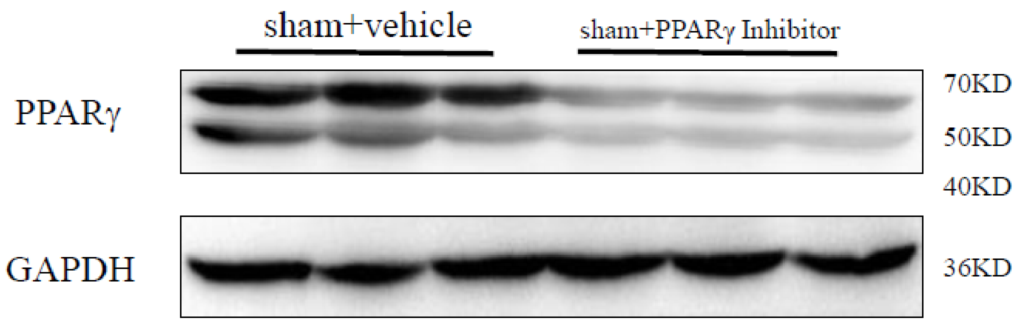

Figure 6B

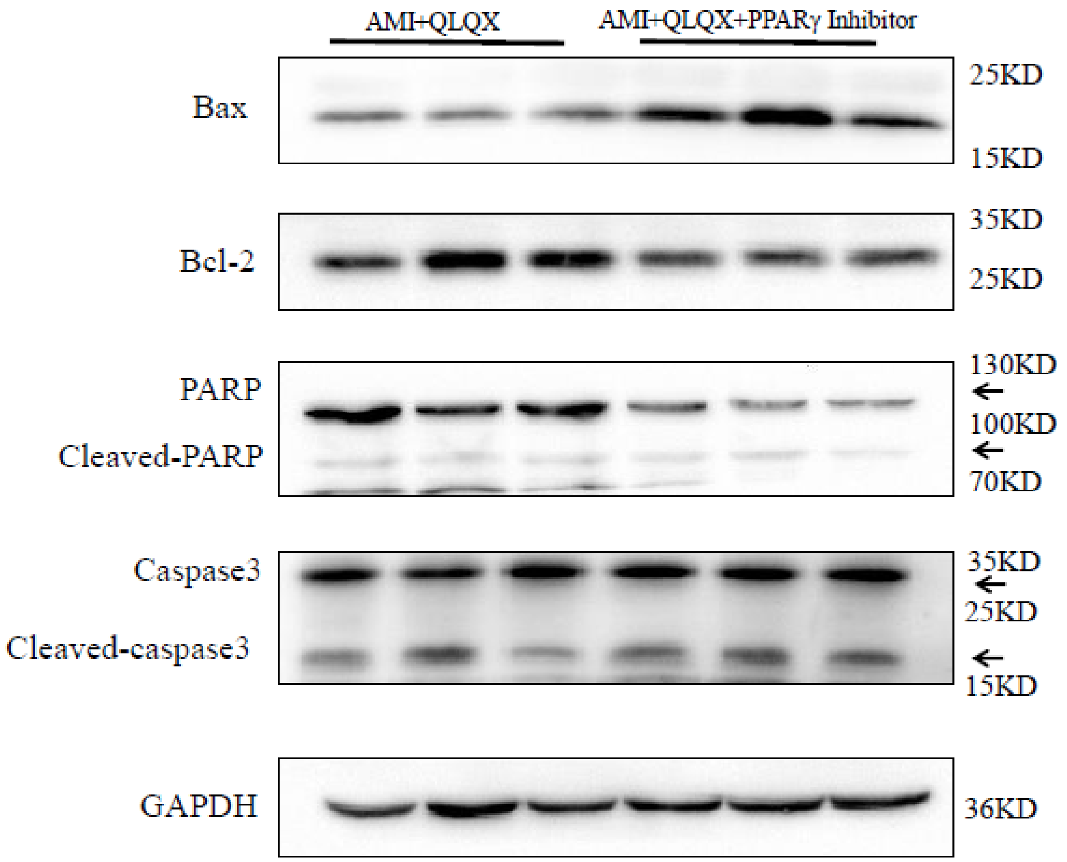

Supplemental Figure 2

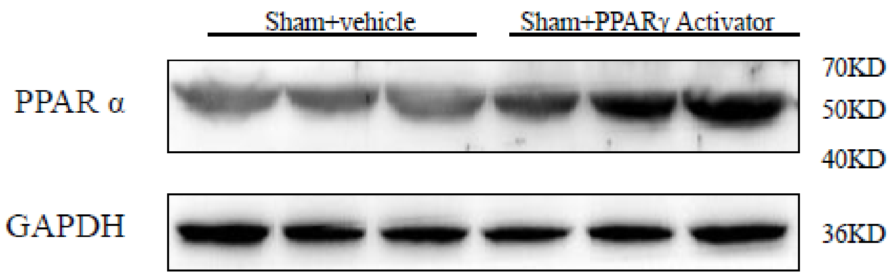

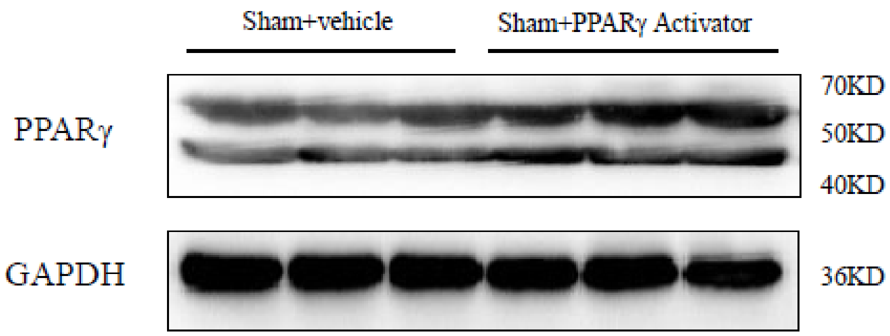

Supplement: Supplementary Information — Supplementary Material [file srep08374-s1.pdf]
